# Value of sarcopenia in the resection of colorectal liver metastases—a systematic review and meta-analysis

**DOI:** 10.3389/fonc.2023.1241561

**Published:** 2023-09-26

**Authors:** D. Wagner, V. Wienerroither, M. Scherrer, M. Thalhammer, F. Faschinger, A. Lederer, H. M. Hau, R. Sucher, P. Kornprat

**Affiliations:** Division for General, Visceral, and Transplantation Surgery, Department of Surgery, Medical University of Graz, Graz, Austria

**Keywords:** colorectal liver metastases, colorectal cancer, liver metastases, overall survival, disease free survival, sarcopenia

## Abstract

**Introduction:**

Sarcopenia is defined as a decline in muscle function as well as muscle mass. Sarcopenia itself and sarcopenic obesity, defined as sarcopenia in obese patients, have been used as surrogates for a worse prognosis in colorectal cancer. This review aims to determine if there is evidence for sarcopenia as a prognostic parameter in colorectal liver metastases (CRLM).

**Methods:**

PubMed, Embase, Cochrane Central, Web of Science, SCOPUS, and CINAHL databases were searched for articles that were selected in accordance with the PRISMA guidelines. The primary outcomes were overall survival (OS) and disease-free survival (DFS). A random effects meta-analysis was conducted.

**Results:**

After eliminating duplicates and screening abstracts (*n* = 111), 949 studies were screened, and 33 publications met the inclusion criteria. Of them, 15 were selected after close paper review, and 10 were incorporated into the meta-analysis, which comprised 825 patients. No significant influence of sarcopenia for OS (odds ratio (OR), 2.802 (95% confidence interval (CI), 1.094–1.11); *p* = 0.4) or DFS (OR, 1.203 (95% CI, 1.162–1.208); *p* = 0.5) was found, although a trend was defined toward sarcopenia. Sarcopenia significantly influenced postoperative complication rates (OR, 7.905 (95% CI, 1.876–3.32); *p* = 0.001) in two studies where data were available.

**Conclusion:**

Existing evidence on the influence of sarcopenia on postoperative OS as well as DFS in patients undergoing resection for CRLM exists. We were not able to confirm that sarcopenic patients have a significantly worse OS and DFS in our analysis, although a trend toward this hypothesis was visible. Sarcopenia seems to influence complication rates but prospective studies are needed.

## Introduction

Colorectal cancer (CRC), with 1.8 million new cases diagnosed per year, has been found to be the fourth most incident cancer worldwide. It accounts for the second-most cancer-related deaths worldwide, which means 800,000 CRC-related deaths annually (1, 2).

Colorectal liver metastases (CRLM) are present in approximately 15% of CRC patients at the time of the primary diagnosis, and another 16% of patients develop CRLM throughout their 5-year follow-up after CRC treatment (3, 4). With a 5-year survival of about 16% of overall CRLM patients, this number increases in resectable situations to up to 50% (5).

Various risk factors for the development of CRC have been defined. One of the major risk factors appointed by the World Health Organization is obesity with a body mass index (BMI) above 30 kg/m^2^ for adults (6, 7). It has been well described, and the rise of obesity in all industrial nations worldwide might also contribute to the higher incidences of CRC in these industrial populations (8, 9).

For obese patients, worse overall survival as well as higher incidences of CRLM and worse outcomes after CRLM resection has been shown, rebutting the “obesity paradox,” which had been described earlier, stating that moderate obesity might even be protective for patients sustaining CRC (10, 11). However, there is still limited high-quality evidence on the real impact of obesity on perioperative as well as long-term outcomes after resection for CRLM.

Sarcopenia has been used as a surrogate for muscle wasting in previous years and is defined as a progressive and generalized skeletal muscle disorder that is associated with an increased likelihood of adverse outcomes (12, 13). It comprises not only a decline in muscle mass but also a decline in muscle function. Generally, sarcopenia has been found to be present in about 38% of cancer patients at the time of presentation; in CRC patients, about 39% of patients have been described as sarcopenic (14, 15). In metastatic CRC patients, even 44% have been identified in studies as having sarcopenia. On top of over a third of patients being sarcopenic at the time of diagnosis of CRC, treatment of CRC with chemotherapy often leads to a significant reduction in muscle mass on top of already prevalent sarcopenia, leading to CRLM patients, who usually receive chemotherapy prior to resection, offering an even worse premise for a potential resection to the individual patient (16, 17).

Unfortunately, sarcopenia is defined very heterogeneously in the present literature. The definition is mainly based on measures of muscle mass and/or muscle density on computed tomography (CT) imaging. Commonly used measures that have been described are the skeletal muscle index (SMI), the total psoas area (TPA), or the Hounsfield Average Calculation (HUAC). All of these parameters are measured on single cross-sectional CT images of the abdomen at the level of the transverse processes of the third lumbar vertebra (L3), normalized for height. The HUAC reflecting the muscle density is measured using the Hounsfield Units of the psoas muscles in the described images and normalizing the measures for the psoas muscles area. Low muscle density has been used as an indicator for intramuscular adipose tissue content (IMAC) and therefore poorer muscle quality (18–20).

Sarcopenia in obese patients—known as sarcopenic obesity—has emerged in recent years as an additional and sometimes more precise prognostic tool as these patients seem to be highly prone to complications. Sarcopenic obesity has been attributed to poor oncologic as well as surgical prognosis (21, 22).

We aimed to perform a systematic review of sarcopenia in the setting of colorectal liver metastases. The presented review was registered in the PROSPERO database (https://www.crd.york.ac.uk/prospero, ID: 432501).

## Methods

### Search strategy

The search for this review was performed according to the preferred reporting items for systematic reviews and meta-analysis (PRISMA) guidelines (23). The Cochrane Register of Controlled Trials (CENTRAL), Embase, the Web of Science, Medline, and Google Scholar were screened for the search string: “colorectal neoplasms” [MeSH Terms] OR “colorectal neoplasms” [Title/Abstract] OR “colorectal cancer” [Title/Abstract] OR “colorectal carcinoma” [Title/Abstract] OR “colorectal tumor” [Title/Abstract] OR “colorectal adenocarcinoma” [Title/Abstract]) AND (“liver neoplasms” [MeSH Terms] OR “liver neoplasms” [Title/Abstract] OR “liver metastases” [Title/Abstract] OR “hepatic metastases” [Title/Abstract] OR “metastatic liver disease” [Title/Abstract])) AND (“sarcopenia” [MeSH Terms] OR “sarcopenia” [Title/Abstract] OR “muscle wasting” [Title/Abstract] OR “muscle loss” [Title/Abstract] OR “muscle atrophy” [Title/Abstract]. The search was carried out on the 22nd day of May 2023 by Scherrer M and Wagner D.

### Inclusion criteria

Only original studies investigating humans were included in the analysis. Studies were only included if they reported outcomes of patients aged 18 years and above who underwent liver resection with curative intent for colorectal liver metastases or if specific outcomes for sarcopenic patients were reported. Studies that reported a defined outcome as recurrence, disease-free survival, or overall survival were included in further analysis.

Only studies that reported the exact outcome as the main objective, defined as the influence of sarcopenia on patients’ survival and/or recurrence, were selected for further analysis.

### Exclusion criteria

We excluded case series, case reports, reviews, or editorials, as well as experimental research. Only studies written in English were considered for evaluation. Studies reporting from the same databases were limited to the most recent report. We also included outcomes that did not report in numbers and/or missing odds ratios or the possibility of deriving these odds ratios from reported numbers.

### Data sources and study selection

The authors F.F., S.M., and W.D. independently screened titles and abstracts to determine their eligibility for inclusion. Full texts were selected and screened upon identification after abstract reading.

### Data extraction

Eligible studies were selected for further assessment and data extraction. Data were extracted into a database. Data selected included author, publication date, country, number of participants, median age, methods of sarcopenia assessment, preoperative therapy if reported, including chemotherapy, operation method, type of liver resection, and follow-up (duration, reported overall survival or disease-free survival or both as well as perioperative outcomes).

### Quality assessment of included studies and meta-analysis

The quality assessment of the included studies was performed using the Quality in Prognosis Instrument (QUIPS) by three observers (D.W., M.S., and F.F.). The included 10 studies were analyzed using the instrument. The risk of bias was considered low if less than two items were rated as “low risk” or “moderate risk” in the respective assessment categories. Risk of bias was rated “high risk” if more than one item was rated high risk in the respective category (24).

### Statistical analysis

The statistical analysis was performed using SPSS Version 26.0 (SPSS Inc, Chicago, IL, USA). To compare the combined effects of hazard ratios (HR) and/or odds ratios (OR), an inverse approach was applied using 95% confidence intervals (CI) for survival and other outcome data. Heterogeneity was assessed using a random effects model and a C^2^ test with a *p*-value of < 0.1 being considered significant. To assess the quantity of heterogeneity, *I*^2^ statistics were used with a cutoff value of 50%, and odds ratios defined the difference of dichotome variables in the pooled studies (25).

## Results

### Description of included studies

**Figure 1:**
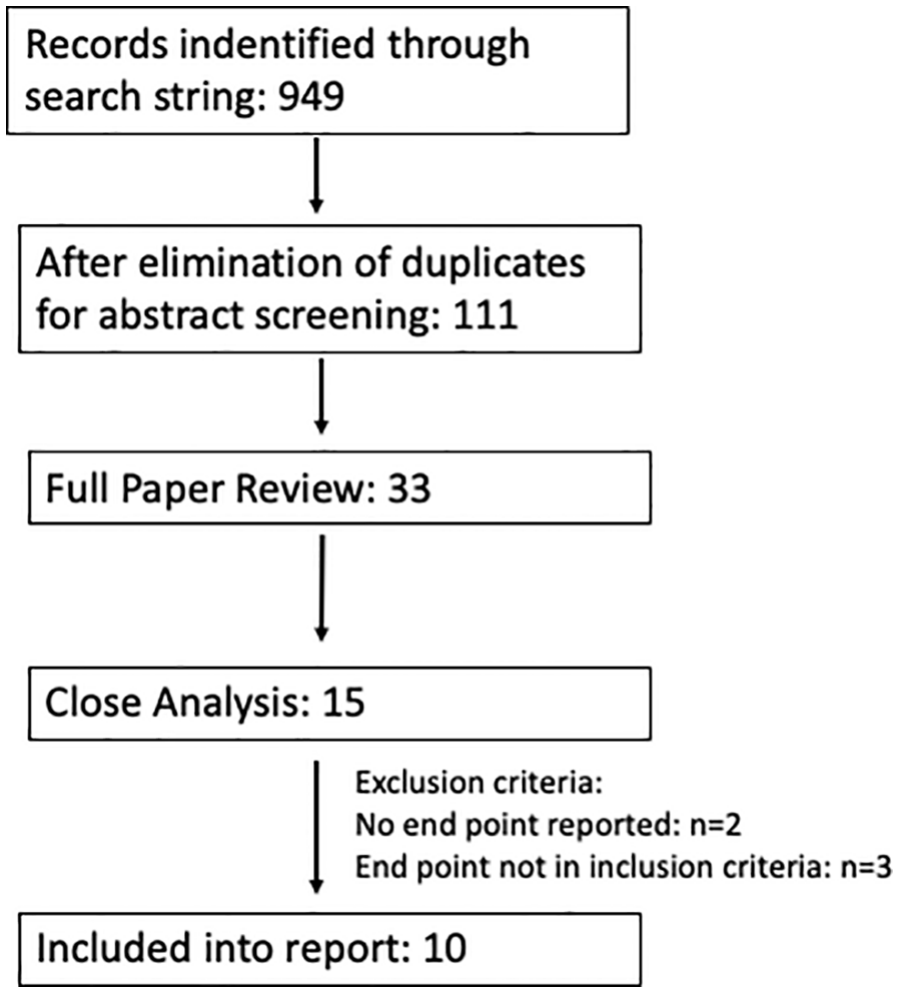
PRISMA flow chart of the search for the review.

The initial search led to 949 studies. After the removal of duplicates (*n* = 838), 111 studies were screened, and a further 78 studies were excluded after abstract screening. The full publications of the remaining 33 studies were screened, and 15 were selected for inclusion into a close assessment. After an independent full-paper review by two investigators, five studies were excluded (no reporting of endpoints *n* = 2, reporting of endpoints not in inclusion criteria *n* = 3). The PRISMA Flow Chart is depicted in **Figure 1**.

So our search derived 10 studies with 1,619 participants that were included in the analysis. Studies were published between 2012 and 2022 and were published by Asian (*n* = 3), European (*n* = 6), and one US American study group (26–35). All studies were retrospective in nature. Databases for the reports had been compiled in a median of 108 months (range: 48–132 months) and mostly included all recipients who underwent CRLM resection in the respective centers and who had undergone preoperative imaging via CT scans, including the lumbar vertebral area at the level of L3. Only Yang et al. included patients who had undergone neoadjuvant treatment and therefore only investigated a limited number of patients who had undergone hepatic resection in their respective centers (33). The characteristics of the included studies are compiled in **Table 1**.

**Table 1:** All charactericstis of the included studies.

Author	Year	Country	Sample size	Study design	Sarcopenia assessment	Cutoff values	Cutoff definition
Erikkson et al.	2017	Sweden	97	Retrospective	SMI	Males: 52.4 cm^2^/m^2^ Females: 38.5 cm^2^/m^2^	Prado et al.
Vledder et al.	2012	The Netherlands	196	Retrospective	HU adipose tissue and skeletal muscle mass	Lowest quartile (sex-specific)	Statistical stratification
Kobayashi et al.	2018	Japan	124	Retrospective	SMI	Lowest quartile (sex spcific)	Stastitical stratification
Bajric et al.	2022	Austria	355	Retrospective	SMI	Males: 52.4 cm^2^/m^2^ Females: 38.5 cm^2^/m^2^	Prado et al.
Lodewick et al.	2015	The Netherlands	171	Retrospective	SMI	Males: 43 cm^2^/m^2^ if BMI < 25; 53 cm^2^/m^2^ if BMI > 25 Females: 41 cm^2^/m^2^	Martin et al.
Runkel et al.	2021	Germany	94	Retrospective	SMI	Males: 52.4 cm^2^/m^2^ Females: 38.5 cm^2^/m^2^	Prado et al.
Liu et al.	2022	China	182	Retrospective	HUAC	HUAC < 22	Statistical stratification
Pessia et al.	2021	Italy	74	Retrospective	SMI	Males: 43 cm^2^/m^2^ if BMI < 25; 53 cm^2^/m^2^ if BMI > 25 Females: 41 cm^2^/m^2^	Martin et al.
Yang et al.	2023	China	67	Retrospective	SMI	Males: 52.4 cm^2^/m^2^ Females: 38.5 cm^2^/m^2^	Prado et al.
Peng et al.	2011	USA	259	Retrospective	TPA	500 mm^2^/m^2^	Optimum stratification

Study participation, sarcopenia measurement and cutoffs as well as reference for chosen cutoffs are outlined as is the year of publication stratified by the respective first author. SMI, skeletal muscle index; TPA, total psoas area; HU, Hounsfield units; HUAC, Hounsfield units average calculation.

Six of the included studies reported primary tumor location (26–28, 31, 32, 34), and seven reported neoadjuvant and/or adjuvant chemotherapy as confounders (26–29, 33–35). All patients included underwent liver resection, whereas only five studies stated the resection technique (26, 27, 31, 33, 35), and only one reported the operation method in detail (35).

Sarcopenia was identified to be prevalent in 825 patients in all studies. Baseline characteristics were outlined in nine of 10 studies included in the analysis according to nonsarcopenic and sarcopenic patients. In four of them, baseline characteristics differed significantly in age, BMI, adipose tissue, tumor markers, tumor location, and the neutrophil-to-lymphocyte ratio (26, 28, 29, 34). Yang et al. also focused on the progression of sarcopenia after neoadjuvant chemotherapy (33).

### Confounder and outcome assessment

Confounders assessed along with sarcopenia differed widely throughout the studies. All studies used age, BMI, tumor stage with TNM classification, ASA status, and gender as confounding variables. The outcome assessment in nine studies was defined as overall survival as well as disease-free survival (26–32, 34). Both were defined homogeneously throughout the nine studies as overall patient survival being the patient survival in the respective follow-up and disease-free survival being the recurrence-free survival in the respective follow-up. Only three studies (Runkel et al., Bajric et al., and Peng et al.) used postoperative morbidity and mortality as combined outcome endpoints, defining patients’ 30-day morbidity using Clavien–Dindo classification (26, 31, 35). Of them, only Bajric and Peng et al. could be used for meta-analysis, as the difference between the sarcopenic and nonsarcopenic patients was not stated in Runkel et al., neither in number nor in statistical form (26, 31).

### QUIPS checklist

In the quality assessment using the QUIPS chart, five studies were rated as having an overall low risk for bias, three had a moderate bias risk and two showed a high risk for bias in the category study participation/selection of study participants and assessment for confounders. All results are compiled in **Table 2**.

**Table 2:** Data of the QUIPS assessment of the included studies.

Author	Study participation	Study attrition	Prognostic factor measurement	Study confounding	Outcome measurements	Statistical analysis and reporting
**Erikkson et al.**	Moderate risk	Moderate risk	Low risk	Low risk	Low risk	Low risk
**Vledder et al.**	High risk	Moderate risk	Moderate risk	Moderate risk	Low risk	Low risk
**Kobayashi et al.**	Low risk	Moderate risk	Low risk	Moderate risk	Low risk	Moderate risk
**Bajric et al.**	Low risk	Moderate risk	Low risk	Moderate risk	Low risk	Low risk
**Lodewick et al.**	Moderate risk	Moderate risk	Moderate risk	Moderate risk	Low risk	Low risk
**Runkel et al.**	Moderate risk	Moderate risk	Low risk	Low risk	Low risk	Moderate risk
**Liu et al.**	Moderate risk	Moderate risk	Low risk	Moderate risk	Low risk	Low risk
**Pessia et al.**	Moderate risk	Moderate risk	Moderate risk	High risk	Low risk	Moderate risk
**Yang et al.**	Moderate risk	Moderate risk	Low risk	Low risk	Low risk	Low risk
**Peng et al.**	Moderate risk	Moderate risk	Moderate risk	Moderate risk	Low risk	Moderate risk

### Sarcopenia assessment and definition

The definition of sarcopenia was very heterogeneous throughout the studies. Most of the included studies used the skeletal muscle index (SMI) to define sarcopenic patients (*n* = 7); four of these studies defined sarcopenia according to the established cutoffs by Prado et al., and two defined sarcopenia using the cutoff values defined by Martin et al. Only one study used statistical stratification to define the cutoffs and used the lowest quartile of their own patient set as a definition for sarcopenia. The other three studies used the total psoas area (TPA) with a cutoff derived by statistical stratification as a definition for sarcopenia, and only two studies incorporated muscle attenuation (i.e., intramuscular adipose tissue) in their primary definition for sarcopenia using the Hounsfield Units as a surrogate for muscle density.

### Meta-analysis of sarcopenia-related outcomes

The studies reported outcomes for a total of 825 patients. Sarcopenic patients therefore comprised 51% of overall patients. Sarcopenia assessment was heterogeneous throughout the studies, with most studies using SMI and cutoffs established by Prado et al. but not all.

**Figure 2:**
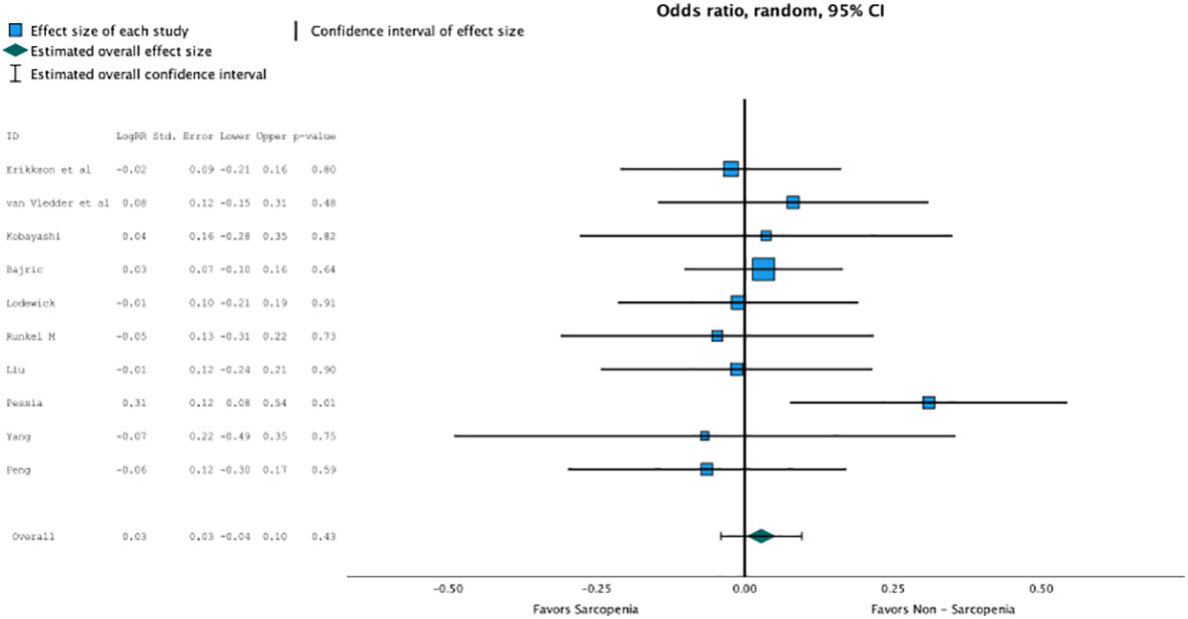
Regression analysis of all aggregated data showed that sarcopenia was associated with post operative overall survival, but no significance was reached due to selective outcome reporting (OR 2.802, CI95%1.094-1.11, p=0.4).

**Figure 3:**
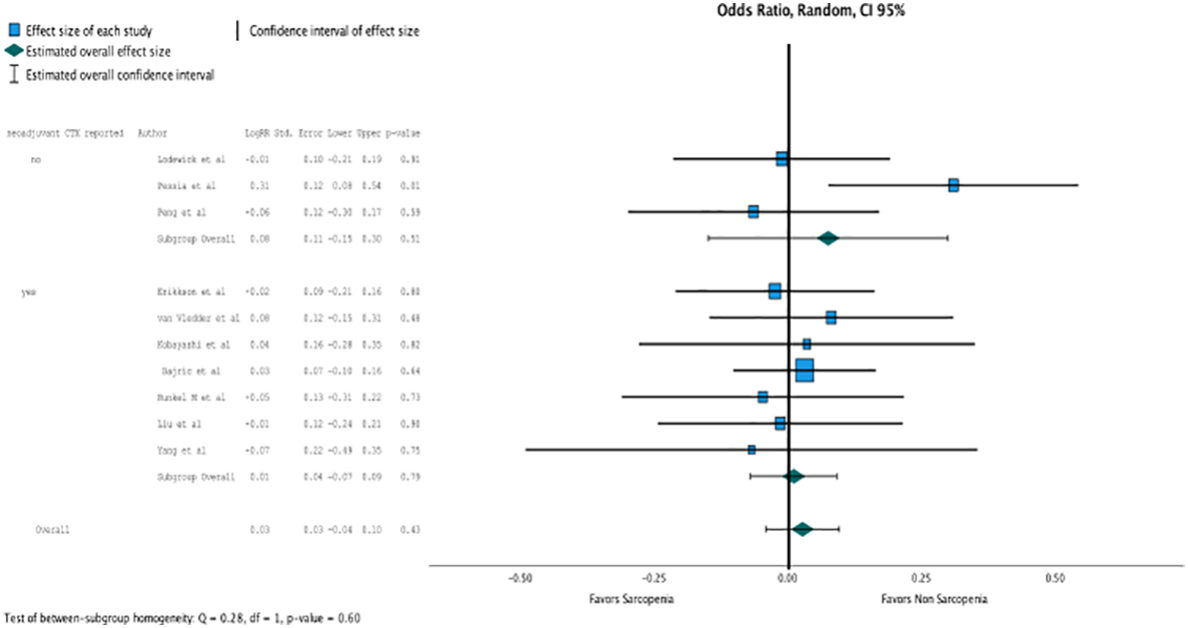
In the subgroup analysis of studies that reported the influence of neoadjuvant therapy vs. studies that did not, no significant influence on overall survival was observed. (OR 2.802, CI95% 1.094-1.11, p=0.4).

Regression analysis of all aggregated data showed that sarcopenia was associated with postoperative overall survival, but no significance was reached due to selective outcome reporting (OR, 2.802 (95% CI, 1.094–1.11); *p* = 0.4, **Figure 2**). In the subgroup analysis of studies that reported the influence of neoadjuvant therapy vs. studies that did not, no significant influence on overall survival was observed, although the death rate recorded as events influenced overall survival, especially in patients with neoadjuvant therapy (OR, 2.802 (95% CI, 1.094–1.11); *p* = 0.4, **Figure 3**). No heterogeneity was found between the studies (*I*^2 =^ 0.28; *p* = 0.6).

**Figure 4:**
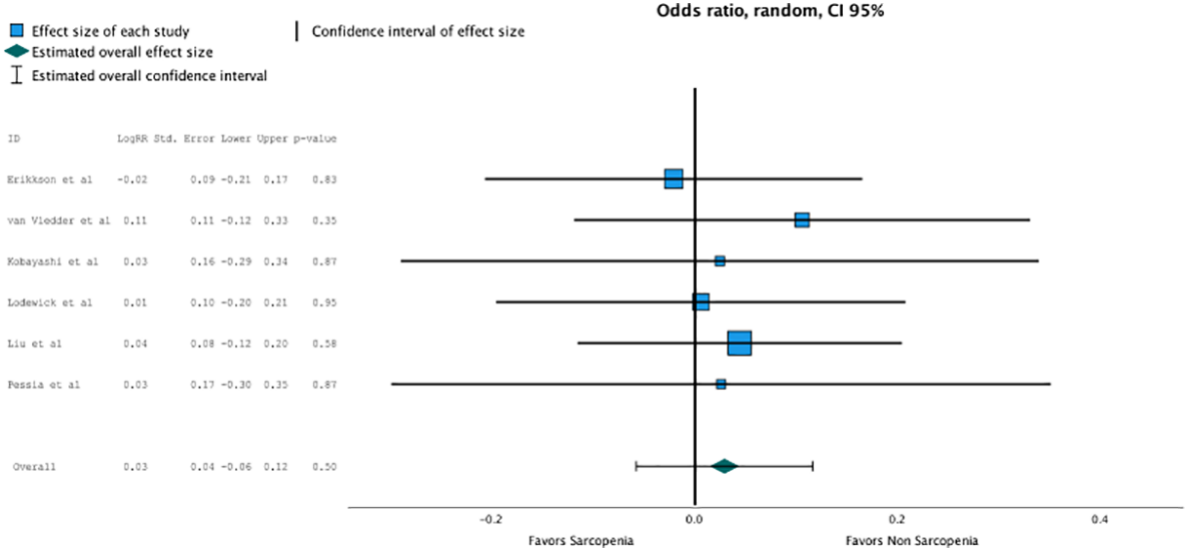
Non sarcopenia seemed better predictive for disease free survival but did not reach statistical significance due heterogenous reporting (OR 1.203, CI95% 1.162-1.208, p=0.5).

Concerning disease-free survival, which was reported in five studies, nonsarcopenia seemed better predictive but did not reach statistical significance due to heterogeneous reporting (OR, 1.203 (95% CI, 1.162–1.208); *p* = 0.5; **Figure 4**).

**Figure 5:**
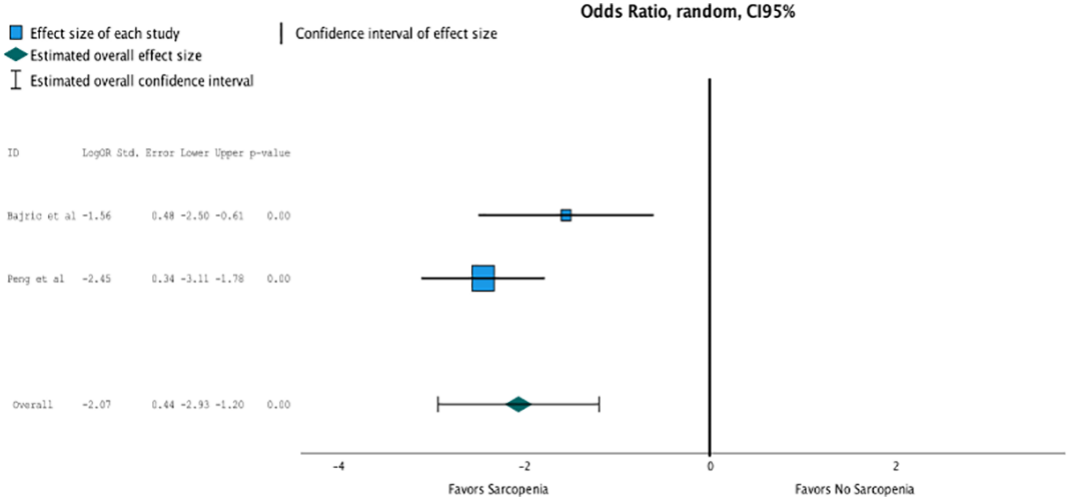
Only two studies reported data on postoperative complications according to sarcopenia. The meta analysis between these studies showed a high association of sarcopenia with postoperative complications (OR 7.905, CI95% 1.876-3.32, p=0.001).

Only two studies reported data on postoperative complications according to sarcopenia. The meta-analysis between these studies showed a high association of sarcopenia with postoperative complications (OR, 7.905 (95% CI, 1.876–3.32); *p* = 0.001; **Figure 5**).

## Discussion

To the best of our knowledge, this is the first meta-analysis that deals with the influence of sarcopenia on the outcome of patients who undergo liver resection for colorectal liver metastases (CRLM) alone. CRLM patients have been incorporated into previous meta-analyses (36, 37). However, due to the unique nature of CRLM and its rising incidence, preoperative assessment is more and more valued in this patient cohort (38). CRLMs are resected in up to 50% of cases. Recent guidelines for liver resection suggest incorporating prehabilitation into their recommendations for preoperative care of patients who undergo liver resection (39). Appropriate sarcopenia diagnosis and knowledge about the impact of sarcopenia on these patients might lead to optimization of preoperative patient care through optimization of prehabilitation and therefore contribute to better postoperative outcomes (19).

Sarcopenia is usually referred to as loss of muscle mass with loss of performance and impaired muscle strength (12, 13, 18). Performing a whole frailty or sarcopenia assessment is time-consuming. This fact often leads to the assessment being omitted or replaced by preoperative image analysis (40, 41). The definition of sarcopenia on preoperative images is still very heterogeneous, both through the working groups and the existing studies (39).

Our review not only confirmed this, but we were also able to display the heterogeneity systematically. Only 40% of the included studies used the same definition for sarcopenia (26, 27, 33, 35), even though 70% of the included studies used the same parameter to assess sarcopenia (*p* = 0.05). This not only is a significant difference; it also depicts the priority most clinicians usually have when it comes to sarcopenia assessment—to do it fast. This again stresses the high need in the clinical setting to have a readily available parameter. Williams et al. recently stressed this need from a perioperative patient management point of view (42). Until a concise and easily assessed parameter is available, sarcopenia will still be treated as a research parameter, although it is associated with dose-limiting toxicity in chemotherapy in other cancer patients (43). This was partially confirmed in the report by Yang et al., who clearly showed that patients who undergo neoadjuvant therapy do experience more pre- and postoperative sarcopenia and concomitantly higher morbidity and mortality rates (33).

Previous meta-analyses have found an association between overall survival and sarcopenia in patients who undergo loco-regional treatment for CRLM (44).

Although a trend toward sarcopenia was associated with lower overall survival rates in our patients, definitive significance was not observed among the included studies. Some studies have already shown this association. For example, Levolger et al. found poorer overall survival in patients undergoing resection of gastrointestnal malignancies.

Unfortunately, in this study, no report exists from an association of CRLM patients. In colorectal patients after resection, sarcopenia has been associated with poorer overall survival in studies by Trejo-Avila et al. This association was not as prominent in our meta-analysis, but it was observed (17).

Trejo-Avila et al. also performed a subanalysis on CRLM resection and postoperative complications. They found only a trend in association. Although we were only able to incorporate two studies into the meta-analysis, our association between sarcopenia and postoperative outcome was more prominent as compared to this previous study (26, 31).

Our analysis did not include studies that incorporated mixed populations due to their heterogenic nature in planning. This might explain this difference in one of our main findings compared to a recent meta-analysis incorporating all liver tumors (37, 45).

However, an association between sarcopenia and postoperative complications, according to Clavien–Dindo, has been reported in different resected tumors—for example, after gastrectomy (OR, 2.17 (95% CI, 1.53–3.08)) (16) or for colorectal cancer (OR, 1.82 (95% CI, 1.36–2.44)) (46). Similar patients with sarcopenia also showed limited survival in patients with pancreatic cancer (OR, 1.80 (95% CI, 1.42–2.29)) or esophageal cancer, as well as an association with higher complication rates (47).

After evaluation of the studies, we feel that the association between sarcopenia and worse postoperative outcomes after CRLM resection is clinically relevant and needs to be evaluated in prospective studies, including prehabilitation protocols prior to liver resection.

Why the association between postoperative complications and sarcopenia is so prominent in our meta-analysis can only be hypothesized. This might also be due to the fact that often multiple metastases are resected in one operation in CRLM patients. All studies that reported postoperative complications in our analysis detailed the complications, with biliary complications and bleeding complications the most prominent of the two studies that could be included in the meta-analysis (26, 31).

There is increased evidence that preoperative prehabilitation in the form of dietary supplements, protein supplementation, and exercise can improve muscle mass, function, and quantity (48). Studies showed that postoperative outcome was improved in patients who underwent resection for gastric cancer, and this is currently under prospective evaluation in these patients (49, 50). Also, chemotherapy tolerance as well as efficacy was ameliorated with the improvement of sarcopenia in recent reports (14, 47, 51).

However, our analysis has several limitations that need to be addressed. All the included studies were retrospective. Until now, no prospective analysis and follow-up of sarcopenic patients who undergo liver resection for CRLM exists. Despite the number of initially screened studies being high, only a limited number of studies could be selected for systematic review, and hence the quality of the analysis might be different in a higher study number setting. Also, the included studies were published over a relatively long period of time, between 2011 and 2023. This was also reflected in the quality of reporting of endpoints and in the quality assessment, with the studies that were reported recently showing lower risks for bias as compared to studies that had been reported earlier. All studies only measured sarcopenia using CT scans, whereas muscle performance seems to be crucial to defining real sarcopenia.

In conclusion, our meta-analysis showed that addressing sarcopenia seems to be beneficial for patients undergoing CRLM resections. A prospective study incorporating sarcopenia as muscle mass and muscle status and incorporating prehabilitation should be performed to accurately assess the value of sarcopenia in the setting of CRLM treatment with and without resection.

## Author contributions

DW, PK, VW, HH, RS: conzeptuatlization of review. DW, MS, FF, VW: search string, literature review, study selection. DW, MS, VW: QUIPS analysis. MS, FF: data extraction. DW, MS: meta analysis. All authors contributed to the article and approved the submitted version.
